# Estimated Glomerular Filtration Rate and Systolic Time Intervals in Risk Stratification for Increased Left Ventricular Mass Index and Left Ventricular Hypertrophy

**DOI:** 10.1097/MD.0000000000002917

**Published:** 2016-03-11

**Authors:** Wen-Hsien Lee, Po-Chao Hsu, Chun-Yuan Chu, Szu-Chia Chen, Hung-Hao Lee, Meng-Kuang Lee, Chee-Siong Lee, Hsueh-Wei Yen, Tsung-Hsien Lin, Wen-Chol Voon, Wen-Ter Lai, Sheng-Hsiung Sheu, Ho-Ming Su

**Affiliations:** From the Graduate Institute of Clinical Medicine (W-HL, S-CC), Faculty of Medicine, College of Medicine (W-HL, P-CH, C-YC, S-CC, C-SL, H-WY, T-HL, W-CV, S-HS, H-MS), Division of Cardiology, Department of Internal Medicine, Kaohsiung Medical University Hospital (W-HL, P-CH, C-YC, H-HL, M-KL, C-SL, H-WY, T-HL, W-CV, W-TL, S-HS, H-MS), and Department of Internal Medicine, Kaohsiung Municipal Hsiao-Kang Hospital, Kaohsiung Medical University, Kaohsiung, Taiwan, ROC (W-HL, S-CC, M-KL, H-MS).

## Abstract

Either decreased renal function or increased systolic time interval is associated with cardiac hypertrophy and poor cardiac outcome. The aim of this study was to evaluate combination of renal function and brachial systolic time intervals were associated with increased left ventricular mass index (LVMI) and left ventricular hypertrophy (LVH).

In total of 990 patients were consecutively included in this study from January 2011 to December 2012. All study participants were further classified into 4 groups by the values of estimated glomerular filtration rate (eGFR) and ratio of brachial preejection period (bPEP) to brachial ejection time (bET). The classification of 4 groups were eGFR ≥ 45 mL/min/1.73 m^2^ and bPEP/bET < 0.38 (group 1), eGFR ≥ 45 ml/min/1.73 m^2^ and bPEP/bET ≥ 0.38 (group 2), eGFR < 45 mL/min/1.73 m^2^ and bPEP/bET < 0.38 (group 3), and eGFR < 45 mL/min/1.73 m^2^ and bPEP/bET ≥ 0.38 (group 4), respectively. Patients in groups 1 and 4 had the lowest and highest LVMI among 4 groups, respectively (*P* < 0.001). In multivariable analyses, increased LVMI and LVH were significantly associated with patients in groups 2, 3 and 4 (vs group 1) (*P* ≤ 0.019).

Our study demonstrated that joined parameter of renal function and systolic time intervals, in terms of eGFR and bPEP/bET, might be an alternative method in risk stratification for increased LVMI and LVH.

## INTRODUCTION

Abnormal cardiac and vascular structure and function are increased the risk of cardiovascular mobility and mortality.^1^ Most studies pay much attention to left ventricular hypertrophy (LVH) because of development of heart failure and high cardiovascular death.^2–4^ The mechanisms associated with LVH are multifactors. Either cardiac volume or pressure overload triggers several biological signal cascades leading to cardiac hypertrophy.^2^ Meanwhile, either arterial pressure or fluid volume overload are common noted in patients with chronic kidney disease (CKD), which can contribute to abnormal cardiac geometry and function in these patients. In epidemiological studies, advanced CKD patients had a high prevalence of LVH.^5,6^ The prevalence of LVH is inversely proportional to the value of eGFR.^5^ CKD patients, who either have reduced estimated glomerular filtration rate (eGFR) or renal damage, frequently have volume retention and electrolyte imbalance which may cause abnormal cardiac function, increased left ventricular mass index (LVMI), and adverse cardiac events. Prior studies demonstrated that reduced eGFR was the important factor affecting not only the progression of renal disease but also abnormal left ventricular geometry.^7,8^

On the opposite hand, preexisting cardiac disease is also associated with renal damage and rapid deterioration of eGFR.^9^ The relationship between renal and cardiac disease is close and bidirection.^10^ Parameters of cardiac systolic function can be measured from echocardiography and peripheral arterial Doppler waveform.^11–13^ Systolic time intervals, including preejection period and ejection time, are a well-known indicator for global cardiac systolic function.^14^ Brachial preejection period (bPEP) and brachial ejection time (bET) calculated from waveform of brachial arterial pressure, electrocardiogram and phonocardiogram were alternative parameters for evaluation of left ventricular systolic function.^14^ In our previous studies, prolonged bPEP, short bET, and high bPEP/bET were associated with cardiac systolic dysfunction.^12,15^ Furthermore, bPEP/bET can serve as a useful parameter in prediction of LVH and cardiac death in patients with CKD and hemodialysis.^15–17^

Although deterioration of renal function and brachial systolic time internals, in terms of decreased of eGFR and high bPEP/bET, have significant associated with LVH, there was no study investigated LVH from combining these 2 important indicators. We hypothesized that mixed parameters of renal function and brachial systolic time intervals were useful tools in the risk classification for LVH. The goal of our study was to evaluate the role of joined with eGFR and bPEP/bET in risk categorization for increased LVMI and LVH.

## METHODS

### Study Subjects and Design

**FIGURE 1:**
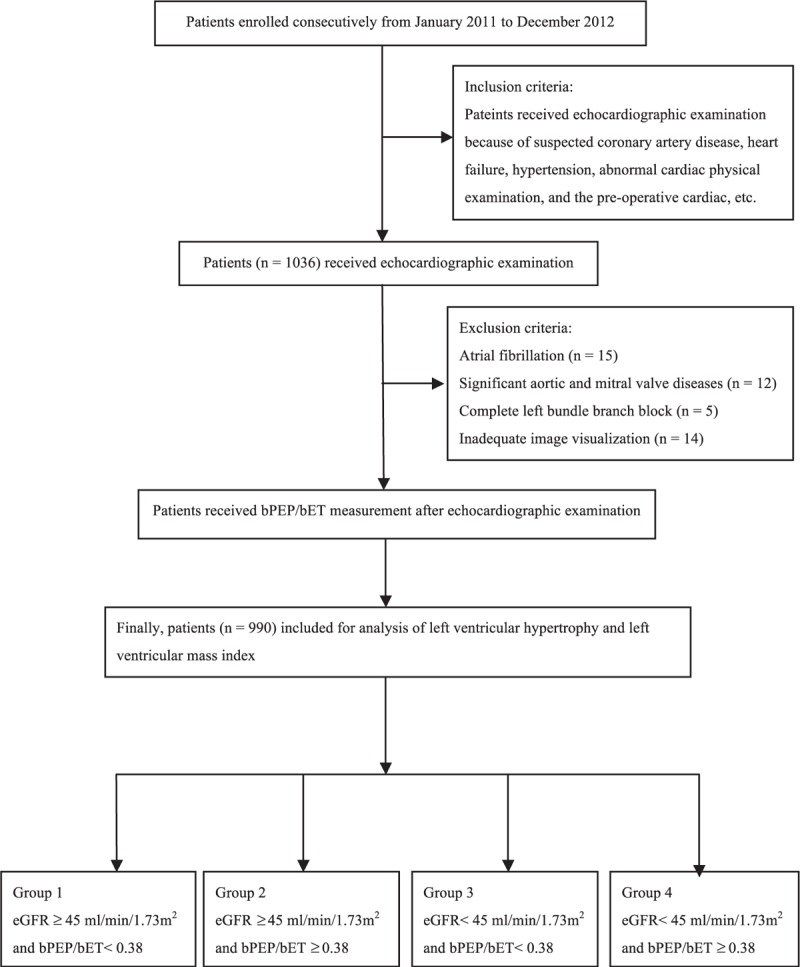
Flow chart of study patients. bPEP/bET = brachial preejection period/brachial ejection time; eGFR = estimated glomerular filtration rate.

Study participants were enrolled for echocardiographic survey in a regional hospital in Taiwan owing to suspected cardiovascular disease from January 2011 to December 2012 (Figure 1). Study participants were excluded due to atrial fibrillation, significant valvular heart disease, complete left bundle branch block, or poor image visualization. Finally, total 990 patients were enrolled for echocardiographic and bPEP/bET examination. The study design was approved by the institutional review board of the Kaohsiung Medical University Hospital (KMUH-IRB-20140256).

### Echocardiographic Assessment

All study patients were received echocardiographic examination by a standard protocol.^12,18^ A single experienced cardiologist performed all echocardiographic examination and acquired image using the Vivid 7 (General Electrics, Horten, Norway). From standard transthoracic view, we measured left ventricular internal diameter (LVID), interventricular septal wall thickness (IVST), and left ventricular posterior wall thickness (LVPWT) in the end left ventricular diastolic phase. The Doppler and tissue Doppler parameters, such as transmitral E wave velocity (E), E-wave deceleration time, transmitral A wave velocity, and early diastolic mitral velocity (Ea), were measured from standard apical four-chamber view.^18^ We calculated left ventricular ejection fraction (LVEF) and Left ventricular mass by the modified Simpson method and the Devereux-modified method, respectively.^19^ LVMI was calculated by dividing left ventricular mass by body surface area. In the present study, we defined LVH as LVMI more than 115 g/m^2^ in men and more than 95 g/m^2^ in women.^18^ All echocardiographic parameters were acquired from 3 continued beats and measured from offline EchoPAC software by a single experienced cardiologist.

### Measurement of Blood Pressures, bPEP, and bET

All blood pressures, bPEP, and bET measurements were obtained after echocardiographic examination. The bPEP, bET, and ratio of bPEP/bET were calculated from an ABI-form device (VP1000) by a standard measurement.^12,20^

### Collection of Medical Characteristics and Laboratory Data

Baseline medication, personal characteristic, and laboratory data were collected from medical records. The value of eGFR was calculated by the equation of Modification of Diet in Renal Disease study.^21^

### Classification of Study Population

The study participants were divided into 4 groups by the values of eGFR and bPEP/bET. Patients were classified into 4 groups when their eGFR ≥ 45 mL/min/1.73 m^2^ and bPEP/bET < 0.38 (group 1), eGFR ≥ 45 mL/min/1.73 m^2^ and bPEP/bET ≥ 0.38 (group 2), eGFR < 45 mL/min/1.73 m^2^ and bPEP/bET < 0.38 (group 3), and eGFR < 45 mL/min/1.73 m^2^ and bPEP/bET ≥0.38 (group 4).^12^

### Statistical Analysis

Statistical analysis was calculated by SPSS 18.0 (SPSS, Inc., Chicago, State of Illinois). The baseline, laboratory, and echocardiographic data were presented as percentage or mean ± standard deviation. Four study groups were analyses by 1-way analysis of variance and Bonferroni post hoc test. Among study groups, group 1 was taken as reference category. Variables which were significance in univariate analysis were selected into multivariable linear and logistic analyses for determinants of LVMI and LVH, respectively. The statistical difference was considered when the *P*-value < 0.05.

## RESULTS

A total of 990 patients were divided into 541, 236, 142, and 71 patients in groups 1, 2, 3, and 4, respectively. The differences of clinical data and echocardiographic parameters among four study groups are shown in Tables 1 and 2. Compared with patients in group 1 (LVMI = 126.6 ± 33.4 g/m^2^, LVH = 69.8%), patients in group 2 (LVMI = 141.6 ± 45.8 g/m^2^, LVH = 74.2%), group 3 (LVMI = 148.5 ± 45.2 g/m^2^, LVH = 89.4 %), and group 4 (LVMI = 181.9 ± 57.1 g/m^2^, LVH = 88.7%) had a higher LVMI (*P* < 0.001) and higher prevalence of LVH (*P* ≤ 0.041). Table 3 shows the determinants of LVMI in all study participants by univariate and multivariate analyses. In the univariate analysis, age, male gender, smoking, diabetes, hypertension, cerebrovascular disease, systolic blood pressure, body mass index, using of angiotensin II receptor blockers (ARBs), β-blockers, calcium channel blockers (CCBs) and diuretics, triglyceride and groups 2, 3, and 4 (vs group 1) were significantly associated with LVMI. In the multivariate analysis, increased LVMI were independently associated with old age, male gender, smoking, high systolic blood pressure, increased body mass index, using of CCBs and diuretics, group 2 (unstandardized coefficient β = 10.364, *P* = 0.001), group 3 (unstandardized coefficient β = 10.670, *P* = 0.007) and group 4 (unstandardized coefficient β = 42.257, *P* < 0.001). Table 4 shows the determinants of LVH in all participants by univariate and multivariate analyses. In the univariate analysis, age, male gender, diabetes, hypertension, systolic blood pressure, heart rate, body mass index, use of ARBs, β-blockers, CCBs and diuretics, and groups 3 and 4 (vs group 1) were significantly associated with LVH. In the multivariate analysis, LVH was independently associated with old age, male gender, high systolic blood pressure, rapid heart rate, increased body mass index, using of diuretics, group 2 (odds ratio = 1.602, *P* = 0.017), group 3 (odds ratio = 2.094, *P* = 0.019), and group 4 (odds ratio = 3.213, *P* = 0.006).

**TABLE 1:**
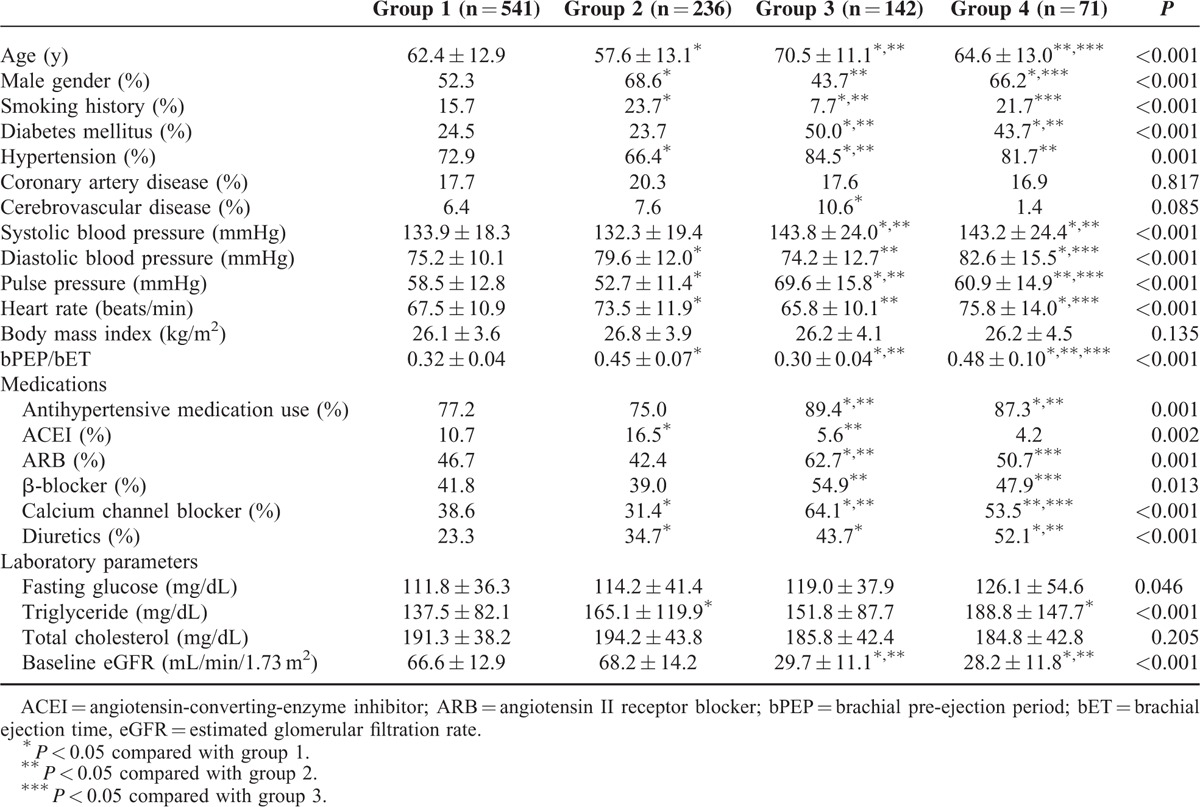
Clinical Characteristics of Patients Among Study Groups

**TABLE 2:**
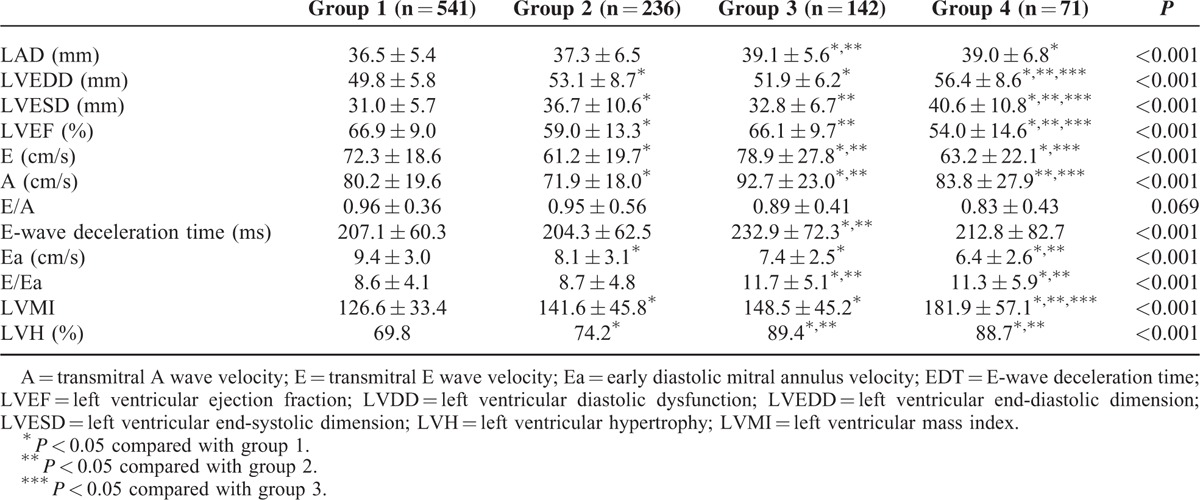
Echocardiographic Characteristics of Patients Among Study Groups

**TABLE 3:**
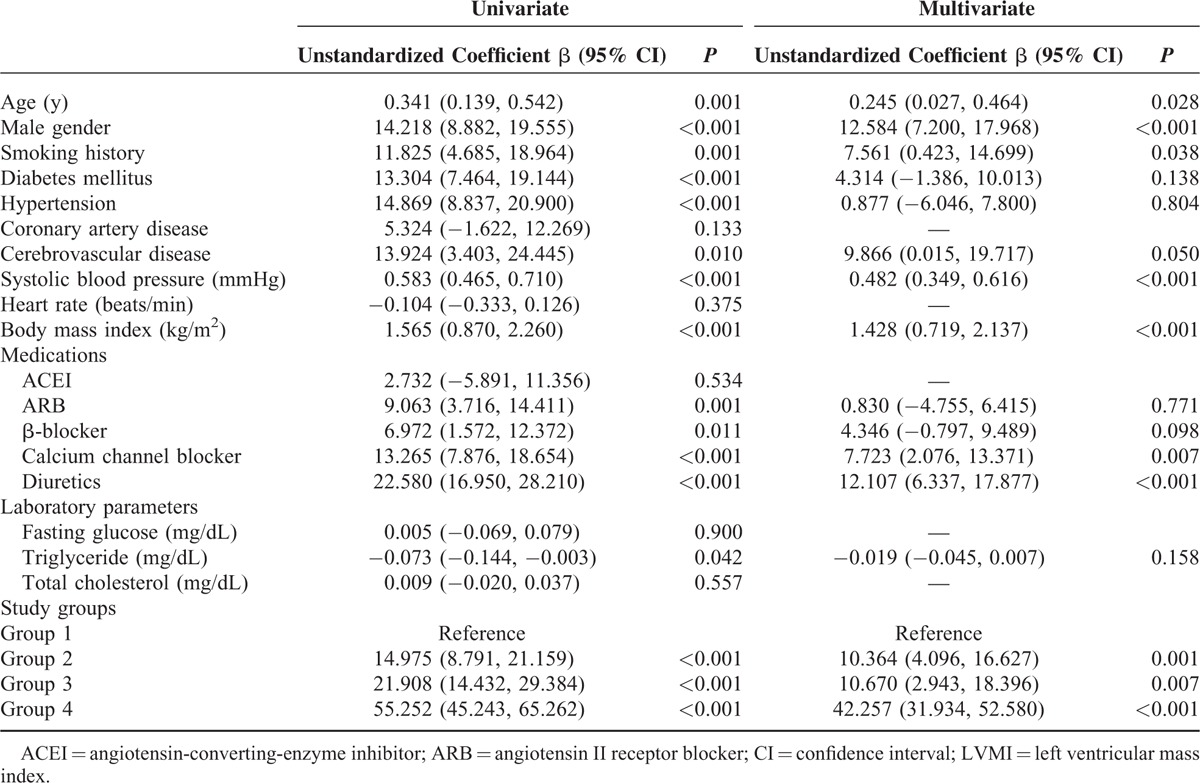
Determinants of LVMI in Study Subjects

**TABLE 4:**
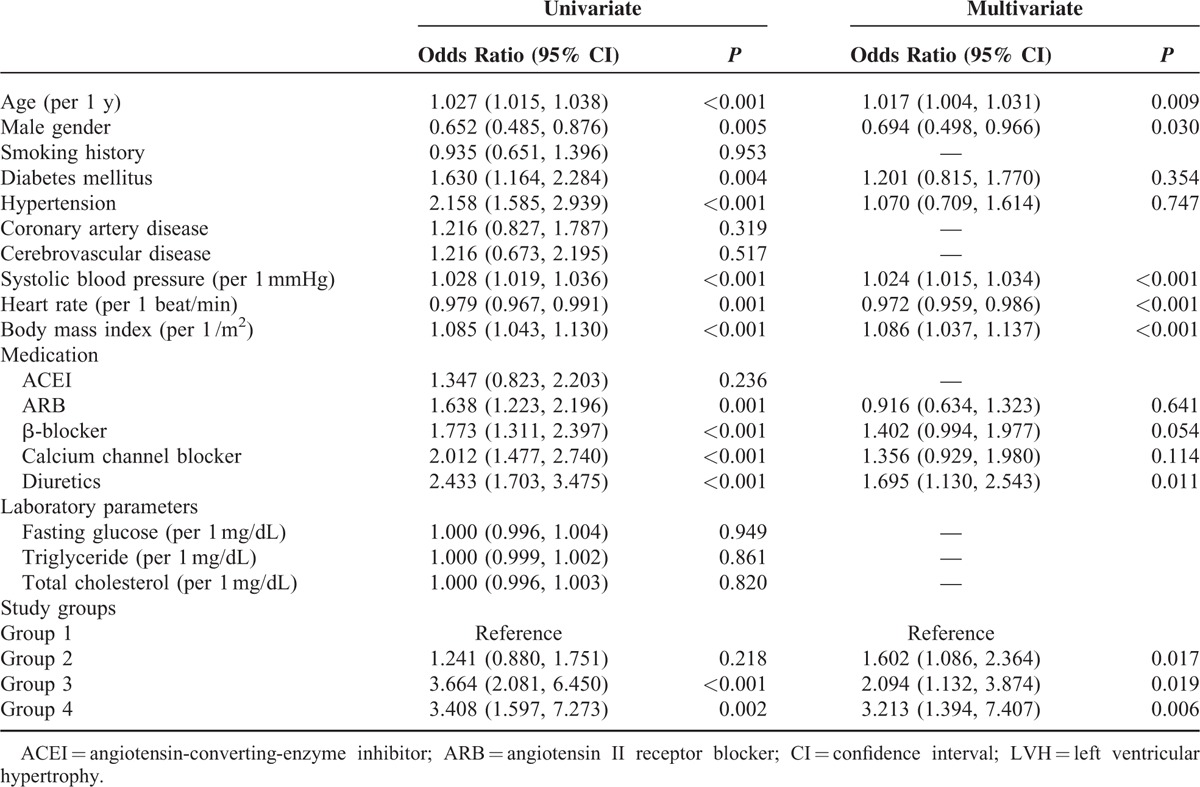
Determinants of LVH in Study Subjects

## DISCUSSION

In the present study, we demonstrated that combination of eGFR and bPEP/bET were useful in risk stratification for increased LVMI and LVH. Patients in group 1 (higher eGFR and lower bPEP/bET) and patients in group 4 (lower eGFR and higher bPEP/bET) had the lowest and highest LVMI among 4 groups, respectively. In addition, compared to patients in group 1, the other groups were associated with a higher LVMI and higher prevalence of LVH in the multivariate analysis.

Boudoulas et al^22^ showed significantly correlation between the ratio of systolic time intervals and LVMI in patients with hypertension. Our previous studies had demonstrated that bPEP/bET was a significant parameter in prediction of decreased LVEF and increased LVMI.^20,23^ The main aim of the present study was to compare LVMI and LVH among 4 groups divided by eGFR and bPEP/bET. Compared with reference group (group 1), we found patients with higher bPEP/bET and/or lower eGFR had an increased LVMI and high prevalence of LVH. These results might suggest patients with relatively normal cardiorenal function had a low LVMI and LVH, but patients with cardiorenal dysfunction had a high LVMI and LVH. Hence, classification of patients into 4 groups using eGFR and bPEP/bET might be useful in recognizing patients with increased LVMI and LVH.

Cardiac and renal failure may interact each other and influence cardiac and renal outcomes.^24^ Mechanisms of cardiorenal interaction may include impaired endothelial function, anemia, systemic inflammation, increased active oxygen species or activation of adrenergic nervous system and rennin–angiotensin system.^24,25^ Pressure and volume overload are frequently noted in CKD patients, which can contribute to LVH and abnormal left ventricular function in these patients. In fact, CKD patients have a high prevalence of increased LVMI and LVH.^5,26^

The causes of cardiac hypertrophy were multifactors. Growth of myocardial mass was influenced by various neurohormones, growth factors, and circulatory cytokines.^27^ High arterial pressure and atherosclerotic process serve important roles in cardiac hypertrophy and its sequentially adverse cardiac events.^28^ Recently, several studies showed genetic factors of hypertension were associated with poor cardiovascular outcome. Lanni et al^29^ showed the glycoprotein IIIa protein with platelet antigen 2 (GPIIIA PlA2) isoform was increased in hypertensive patient with stroke and could serve as a genetic determinant of ischemic stroke among high risk patients. Furthermore, Santulli et al showed the GPIIIA PlA2 polymorphism was an independent predictor for cardiac death (odds ratio = 9.594) in patients with atherosclerotic coronary artery diseases.^30^ Some possible molecular mechanisms, including vascular endothelial dysfunction, sympathetic nervous activation, G-protein-coupled receptor kinase (GRK) expression, and calcium/calmodulin-dependent kinase (CaMK) regulation, were associated with high arterial pressure.^31^ The GRKs family, especially GRK2, played an important role in essential hypertension.^31^ In bench studies, different G-protein-coupled receptors, such as β-adrenergic receptor and angiotensin II type 1A receptor, were regulated by GRK2.^31–33^ Recently, both in vivo and in vitro studies, Sorriento and Cipolletta et al and Ersilia et al demonstrated that GRK2 mediated cardiac hypertrophy through nuclear factor-κB (NFκB) and extracellular regulated kinase pathway, respectively.^34,35^ Besides GRK2, GRK5 also regulated cardiac hypertrophy from NFκB signal transduction.^36^ Additionally, the family of CaMKs and their genes were associated with hypertension and cardiac hypertrophy. Santulli et al demonstrated that CaMK4 gene deletion was an important role in regulation of vascular pressure through inactivation of endothelial nitric oxide synthase.^37^

There were several limitations to our study. First, our study was a cross-sectional design, was performed only in 1 regional hospital and lacked follow up, which limited our study strength. Second, there was no validation of bPEP/bET in patients with atrial fibrillation and valvular heart diseases. Therefore, our results could not be applied in these patients. Finally, we did not check gene polymorphisms in our patients. Hence, the genetic consideration and molecular signal transduction about cardiac hypertrophy were lack in this study.

In conclusion, separating patients into four groups using eGFR and bPEP/bET might be an alternative method in risk stratification for increased LVMI and LVH.
